# Diversity of *Prdm9* Zinc Finger Array in Wild Mice Unravels New Facets of the Evolutionary Turnover of this Coding Minisatellite

**DOI:** 10.1371/journal.pone.0085021

**Published:** 2014-01-13

**Authors:** Jérôme Buard, Eric Rivals, Denis Dunoyer de Segonzac, Charlotte Garres, Pierre Caminade, Bernard de Massy, Pierre Boursot

**Affiliations:** 1 Institute of Human Genetics, UPR 1142, Centre National de la Recherche Scientifique, Montpellier, France; 2 Laboratoire d'Informatique, de Robotique et de Microélectronique de Montpellier, UMR 5506, Université Montpellier 2, Centre National de la Recherche Scientifique, Montpellier, France; 3 Institut des Sciences de l'Evolution Montpellier, Université Montpellier 2, Centre National de la Recherche Scientifique, Institut de Recherche pour le Développement, Montpellier, France; 4 Institut de Biologie Computationnelle, Montpellier, France; National Cancer Institute, United States of America

## Abstract

In humans and mice, meiotic recombination events cluster into narrow hotspots whose genomic positions are defined by the PRDM9 protein via its DNA binding domain constituted of an array of zinc fingers (ZnFs). High polymorphism and rapid divergence of the *Prdm9* gene ZnF domain appear to involve positive selection at DNA-recognition amino-acid positions, but the nature of the underlying evolutionary pressures remains a puzzle. Here we explore the variability of the *Prdm9* ZnF array in wild mice, and uncovered a high allelic diversity of both ZnF copy number and identity with the caracterization of 113 alleles. We analyze features of the diversity of ZnF identity which is mostly due to non-synonymous changes at codons −1, 3 and 6 of each ZnF, corresponding to amino-acids involved in DNA binding. Using methods adapted to the minisatellite structure of the ZnF array, we infer a phylogenetic tree of these alleles. We find the sister species *Mus spicilegus* and *M. macedonicus* as well as the three house mouse (*Mus musculus*) subspecies to be polyphyletic. However some sublineages have expanded independently in *Mus musculus musculus* and *M. m. domesticus*, the latter further showing phylogeographic substructure. Compared to random genomic regions and non-coding minisatellites, none of these patterns appears exceptional. *In silico* prediction of DNA binding sites for each allele, overlap of their alignments to the genome and relative coverage of the different families of interspersed repeated elements suggest a large diversity between PRDM9 variants with a potential for highly divergent distributions of recombination events in the genome with little correlation to evolutionary distance. By compiling PRDM9 ZnF protein sequences in Primates, *Muridae* and Equids, we find different diversity patterns among the three amino-acids most critical for the DNA-recognition function, suggesting different diversification timescales.

## Introduction

In sexually reproducing species, novel combinations of alleles are created at each generation by the process of meiotic recombination. This process of exchange between paternal and maternal chromosomes, called homologs, takes place during the prophase of the first meiotic division. Two types of meiotic recombination events are generated, reciprocal events also called crossovers (COs), and non reciprocal events (NCOs). COs create connections between homologs that are important for the proper segregation of chromosomes at the first meiotic division, and the absence of recombination often leads to sterility [1]. A few sexually reproducing species show the absence of meiotic recombination in one sex, indicating that alternative mechanisms for ensuring a proper reductional segregation and not involving COs, are possible [2]. The process of recombination has consequences on genome diversity and provides a long term advantage where generation of new combinations of alleles enhances the efficiency of natural selection [3], [4]. In addition to new combinations of alleles generated by COs, the process of recombination leads to gene conversion, a non reciprocal exchange of genetic information, which may occur in association or not to COs [5], [6]. Understanding the controls of meiotic recombination both in frequencies and distribution in the genome is thus a major goal for the understanding of how this process ensures a proper chromosome segregation and how it shapes genome diversity [7].

**Figure 1:**
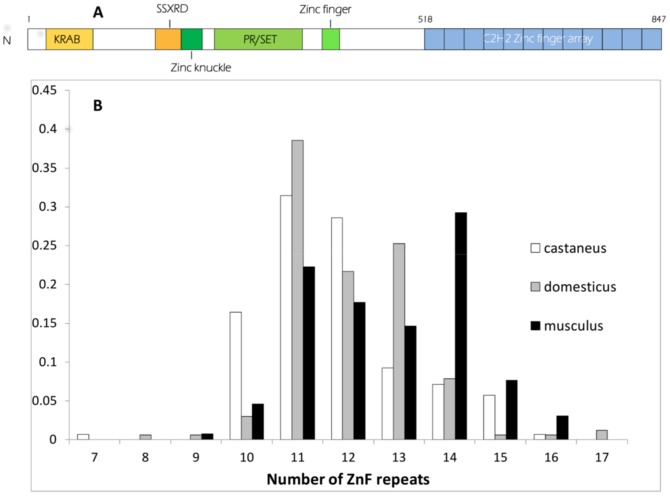
The C2H2 zinc finger domain of PRDM9. (A) PRDM9 contains several identified domains: an amino-terminal region which includes a Krüppel-associated box (KRAB) domain and an SSX repression domain (SSXRD); a PR/SET domain carrying methyltransferase activity, surrounded by a zinc knuckle and a zinc finger; and a long carboxy-terminal C2H2 zinc finger array. In this example, such as observed in the mouse laboratory strain C57BL/6, the array is composed of 12 zinc fingers. (B) Size distribution of the *Prdm9* ZnF arrays (number of ZnF repeats) genotyped in the three subspecies of the house mouse

At the molecular level, meiotic recombination is initiated by the formation of DNA double-strand breaks (DSBs). DSBs have been mapped in various organisms, recently at high resolution in yeast and mice [8], and are catalyzed by the evolutionarily conserved Spo11 protein [9]. In mice and humans, DSB sites are determined by the DNA binding specificity of PRDM9, mediated by a tandem array of C2H2 zinc fingers (ZnFs, Fig. 1A) [10]–[12]. At different human and mouse genomic sites with elevated rates of COs, also called CO hotspots, consensus binding sites for PRDM9 could be detected [13], [14], in a few cases validated by *in vitro* binding assays [10], [15], [16]. Genome wide DSB mapping in mice shows that localization of most DSBs depends on PRDM9 as mice with distinct *Prdm9* alleles with different zinc fingers have essentially no common DSB hotspots [13]. The discovery of blocks of linkage disequilibrium in human populations has demonstrated the existence of hotspots of historical recombination and it was estimated that at least 40% of them may depend on *Prdm9* [14], but further studies suggested that this fraction could be higher [17].

Upon DSB repair, a small region (typically a few ten to hundred base pairs in mammals) around the DSB is replaced by DNA sequences from the homolog, leading to a gene conversion event. The fact that the PRDM9 binding sites are located close to DSBs raises a major question about the evolution of this process. PRDM9 binding sites are thus expected to be often converted upon DSB repair, and mutations within PRDM9 binding sites lowering PRDM9 affinity are expected to be transmitted at higher frequencies, eventually leading to a loss of PRDM9 binding and recombination activity. Comparisons of PRDM9 motifs between humans and chimps show indeed the rapid erosion of DNA motifs associated with PRDM9 activity in humans [11]. Strikingly, a high diversity and rapid evolution of *Prdm9* has been observed in vertebrates, with evidence for positive selection at residues of the zinc fingers involved in DNA recognition [18], [19]. A high diversity of *Prdm9* has also been reported in humans and chimps [20]–[23] and described in laboratory mice [12], [24]. In humans, *Prdm9* allele diversity has been shown to be driven by the high instability of the ZnF tandem array, mostly occurring during meiotic recombination, as demonstrated by sperm typing [25]. In addition, genetic variation at *Prdm9* is involved in hybrid sterility between some strains from two subspecies of the house mouse [26], an indication that the evolution of this gene could drive the evolution of important functions and participate in speciation. Altogether, the evolutionary context of this gene appears relatively complex since it may involve: (i) a high mutation rate due to the minisatellite structure of the ZnF array; (ii) positive, purifying or frequency dependent selection controlling DNA binding specificity; (iii) a runaway process due to the erosion of binding sites, and (iv) epistatic interactions leading to genetic incompatibilities after population divergence.

A first step to understand the evolutionary dynamics of this gene is to describe its natural diversity, which has yet been performed only in humans and to a certain extent chimps and bonobos, and which we undertake here in mice. The house mouse (*Mus musculus*) offers an interesting model because it is made up of three subspecies that have recently radiated from a common ancestor, have colonized distinct distribution areas but are able to exchange genes across several contact zones, despite partial reproductive isolation [27].

We also include for comparison in our study close relatives to the house mouse that are not known to extensively hybridize with the house mouse. We use DNA sequence variation to infer the evolutionary relationships between the ZnF domain alleles, and analyze diversity in relation to the taxonomic and geographic distribution of lineages. We also attempt to detect a relationship between this relatedness of alleles and variations of their function, using as a proxy the distribution in the genome of the DNA patterns they are predicted to recognize according to available *in silico* models. We characterize variations of diversity along the ZnF array in mice. Finally we compile available data on mammals to characterize differences in the constraints on different key amino-acid positions of the PRDM9 ZnFs among lineages.

## Results

### Variation of the number of Zinc finger repeats

We amplified by PCR the ZnF domain of *Prdm9* (Fig. 1A) using primers that flank this region, which consists of tandemly repeated units of 84 nucleotides, each representing a ZnF. We were able to obtain clear PCR products in 250 mice representing several taxa (Table 1 and Table S1 for a summary, Table S2 for a complete list of samples and results). At the resolution of agarose gels, size differences between PCR fragments were compatible with their origin being variations of the number of copies of the 84 bp repeat unit. Size heterozygosities were calculated on the whole sample, which includes mice maintained in laboratory colonies, and exclusively on mice from the field, and the results were comparable (Table 1). In house mice (*Mus musculus*), for which sample sizes are larger, size heterozygosity was not significantly different between the *M. m*. *domesticus* and *M. m*. *castaneus* subspecies (around 50% when considering only samples from the field) and appeared lower in *M. m*. *musculus* (37%, but not significantly lower in either comparison, Fisher's exact test). Expected heterozygosities (*He* in Table 1) were similarly high in the three subspecies (around 80%). Estimates in other taxa are not reported due to limited sample sizes. The number of repeats (potential ZnFs) estimated on the basis of PCR fragment sizes extends from 7 to 17 with the exception of one allele found in a single *M. spretus* that appeared to have only 2 repeats (see below). The distribution of the number of repeats in the three subspecies of the house mouse (Fig. 1B) shows that *M. m. castaneus* tends to have a smaller ZnF domain than *M. m. musculus*, while *M. m. domesticus* shows an intermediate distribution. The distributions are however largely overlapping.

**Table 1:** Repeat copy number variability of *Mus* ZnF *Prdm9* and size heterozygosity.

	All mice				Mice from the field				Nb of repeats	
Taxon	N	NA	H	He	N	NA	H	He	Min	Max
Mus musculus castaneus	70	8	0.486	0.776	62	8	0.484	0.773	7	16
Mus musculus domesticus	83	10	0.410	0.733	56	8	0.500	0.756	8	17
Mus musculus musculus	65	8	0.323	0.803	49	7	0.367	0.793	9	16
Mus musculus molossinus	1	1							14	14
Mus musculus spp.	4	2			4	2			12	13
Mus macedonicus macedonicus	6	6			3	3			9	14
Mus macedonicus spretoides	2	2			2	2			10	11
Mus spicilegus	2	2							8	10
Mus spretus	8	5			2	3			2	13
Mus cypriacus	3	4			3	4			11	15
Mus famulus	1	1							11	11
Mus caroli	2	1							11	11
Mus cervicolor	1	2							8	11
Mus pahari	1	1							13	13
Mus Pyromys platythrix	1	2							13	15

N: number of mice. NA: number of allele sizes. H: observed heterozygosity, He: Expected heterozygosity in panmictic population based on allele frequencies.

### Sequenced samples

We obtained 107 sequences of the *Prdm9* Zinc finger domain from 93 wild mice representing mostly the three subspecies of the house mouse (42 sequences from *M. m. domesticus*, 20 from *M. m. musculus*, 26 from *M. m. castaneus* and 1 from *M. m. molossinus*, the Japanese house mouse that is essentially of *musculus* origin with some contribution from *M. m. castaneus*) but also including representatives of closely related species (7 sequences from *M. spretus*, 6 from *M. macedonicus macedonicus*, 2 from *M. spicilegus*) as well as some more distantly related species (1 *M. famulus*, 1 *M. cervicolor*, 1 *Pyromys plathytrix*). The number of ZnF repeats per sequence ranged from 8 to 17, representing well the size distribution range observed from PCR genotyping. We also added to our dataset the sequences of 6 laboratory strains available in the literature [12], for a total of 113 sequences to be analyzed (Table S1). A complete description of the mice and alleles sequenced can be found in Table S2. All further analyses are based on these 113 sequences, which represented 78 different DNA alleles over the range of the ZnF domain (we will use hereafter numbers 1–78 to designate these alleles, those newly discovered being deposited in GenBank under accession numbers KF462397-KF462503). Note that the strategy we used to obtain the sequences does not allow unbiased estimates of the allele frequency spectrum of the sequence alleles (see Material and Methods). However despite this limitation, it can be said that each of the *M. musculus* subspecies harbors one relatively frequent allele (often found homozygous), at an estimated frequency of 21% for *M. m. domesticus* (allele 46), 26% for *M. m. castaneus* (allele 9) and 30% for *M. m. musculus* (allele 73), and that most other alleles appear much rarer.

### Phylogeny and phylogeography of the alleles

**Figure 2:**
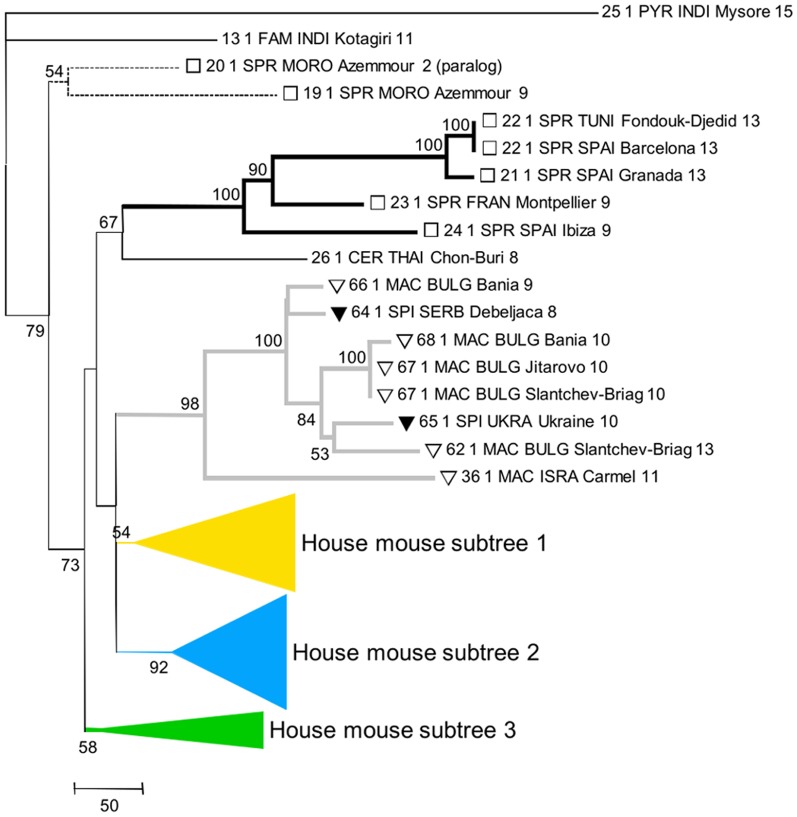
Inferred phylogeny of the *Prdm9* ZnF domain alleles. Taxon names on the branches include allele number, followed by the number of observations, then by taxon code (abbreviation of species name), country code, locality name and number of ZnF repeats. Numbers at the nodes indicate the level of confidence of the node (only values >0.5 reported).

The length variation among *Prdm9* allele sequences, mainly due to ZnF repeat number variations, makes classical alignment methods inappropriate. This hinders chaining a multiple alignment, with phylogeny reconstruction and bootstrapping for inferring an evolutionary tree. Using alignment methods specifically adapted to the minisatellite structure of this genomic region (see Material & Methods), we could reconstruct a tree relating the sequenced alleles (Fig. 2) and observe the relation between taxonomic/geographical origin and evolutionary proximity.

**Figure 3:**
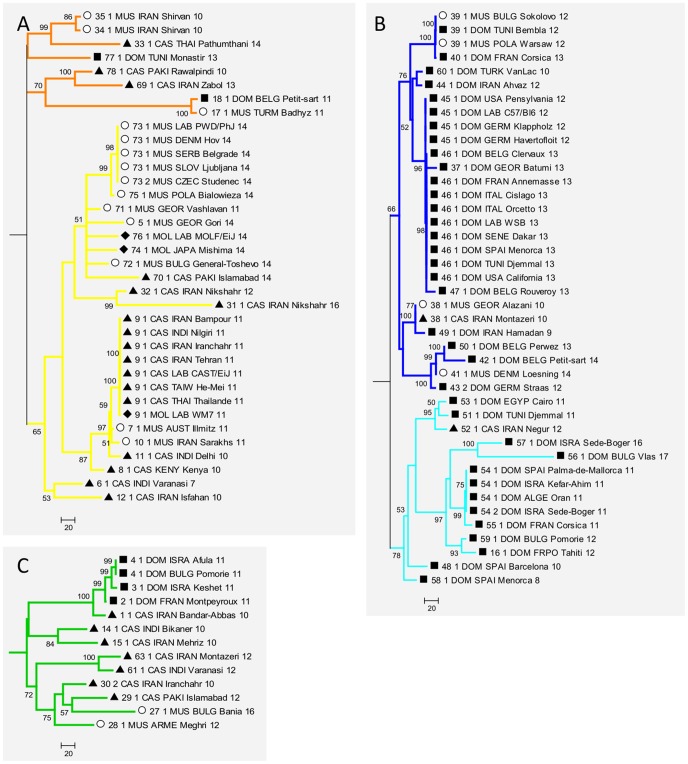
Details of phylogenic tree. The yellow (A), blue (B) and green (C) collapsed parts of the tree in Fig. 2 are expended and represent the different house mouse lineages. Taxon name encoding is as in Fig. 2.

Among the three outgroups to the Palearctic species that we included, *Mus Pyromys platythrix* was expected to be the most distant, followed by *M. cervicolor* and then *M. famulus* [28]. However, the phylogeny of *Prdm9* alleles differs from this expectation in that *M. cervicolor* appears included in the Palearctic. This would deserve further characterization with additional samples of *M. cervicolor*. Among the Palearctic species, most *M. spretus* alleles belong to a well-defined lineage, but alleles 19 and 20 are extremely distant from this group and lie as outgroups to all Palearctic species. The fact that *M. spicilegus* and *M. macedonicus* are grouped together but not separated in the phylogeny is compatible with them being closely related sister species [28]–[31] and would indicate incomplete lineage sorting at the *Prdm9* locus. The house mouse *M. musculus* does not appear monophyletic and thus the phylogeny of the Palearctic group is unresolved. Note though that given the low confidence values at the deep nodes of this part of the tree, we cannot formally exclude that the house mouse is monophyletic. Although few genomic regions have yet been tested, monophyly of the house mouse seems to be the most frequent pattern in the genome (e.g.[32], [33]), with some documented exceptions however (e.g.[33], [34]). All studies based either on a limited number of genomic regions (e.g. [28], [30], [31]) or on genome-wide divergence [29] have failed to resolve the trichotomy between *M. musculus, M. spicilegus-macedonicus and M. spretus*. Extensive incomplete lineage sorting thus appears to have prevailed in this group of taxa, presumably because the differentiation of these three lineages occurred in a short time, thus the lack of resolution of the *Prdm9* phylogeny at this depth is not surprising. Our phylogenetic inference also suggests lineage sharing among subspecies of the house mouse. All of the subtrees we defined (Fig. 2), as well as their subdivisions by color (Fig. 3), contain representatives of more than one subspecies, and several of their well sustained sublineages group together members of at least two of the subspecies. We note however that in the three subtrees of Fig. 3 and at any depth in the trees (except eventually the deepest one), common ancestries group *M. m. domesticus* mice with *M. m. castaneus* mice, or *M. m. musculus* with *M. m. castaneus*, but almost never *M. m. domesticus* with *M. m. musculus*. There are a few exceptions to this rule, but only in cases of shallow connection between alleles, compatible with recent gene flow. These exceptions concern *M. m. domesticus*-like alleles sampled in *M. m. musculus* mice close to hybrid zones between these subspecies (allele 39 in Bulgaria, 38 in Georgia, 41 in Denmark), or further away (allele 39 in Warsaw). Alleles 17 (*M. m. domesticus*, Belgium) and 18 (*M. m. musculus*, Turkmenistan) also break the rule. These few exceptions could be attributed to recent migration across hybrid zones or to recent passive long distance transportation by humans. However, for the older part of the history of house mice (i.e. for deeper nodes in the trees), this observation most probably reflects differences in population sizes between the subspecies, governing patterns of retention of ancestral polymorphisms. There is ample evidence that *M. m. castaneus* is more polymorphic and has higher effective population size than the two other subspecies [32]–[37], which appears to result from higher past and present geographic partition [38], [39]. Among the 27 autosomal loci surveyed [33], a much higher proportion displayed reciprocal monophyly between *M. m. domesticus* and *M. m. musculus* than in the two other pairwise comparisons involving *M. m. castaneus*. In the case of *Prdm9*, monophyly of each subspecies *vis à vis* the two others appears difficult to rigorously assess because many nodes in the tree are poorly resolved, especially the deepest ones. Monophyly seems to be excluded for *M. m. musculus* and *M. m. castaneus* (whose alleles are intermingled in the yellow part of the tree, with reasonable support), but is harder to formally exclude for *M. m. domesticus* if *M. m. castaneus* alleles 38, 52 and 1, that are relatively closely related to groups of *M. m. domesticus* alleles with confidence, are considered recent long range migrants to Iran.

**Figure 4:**
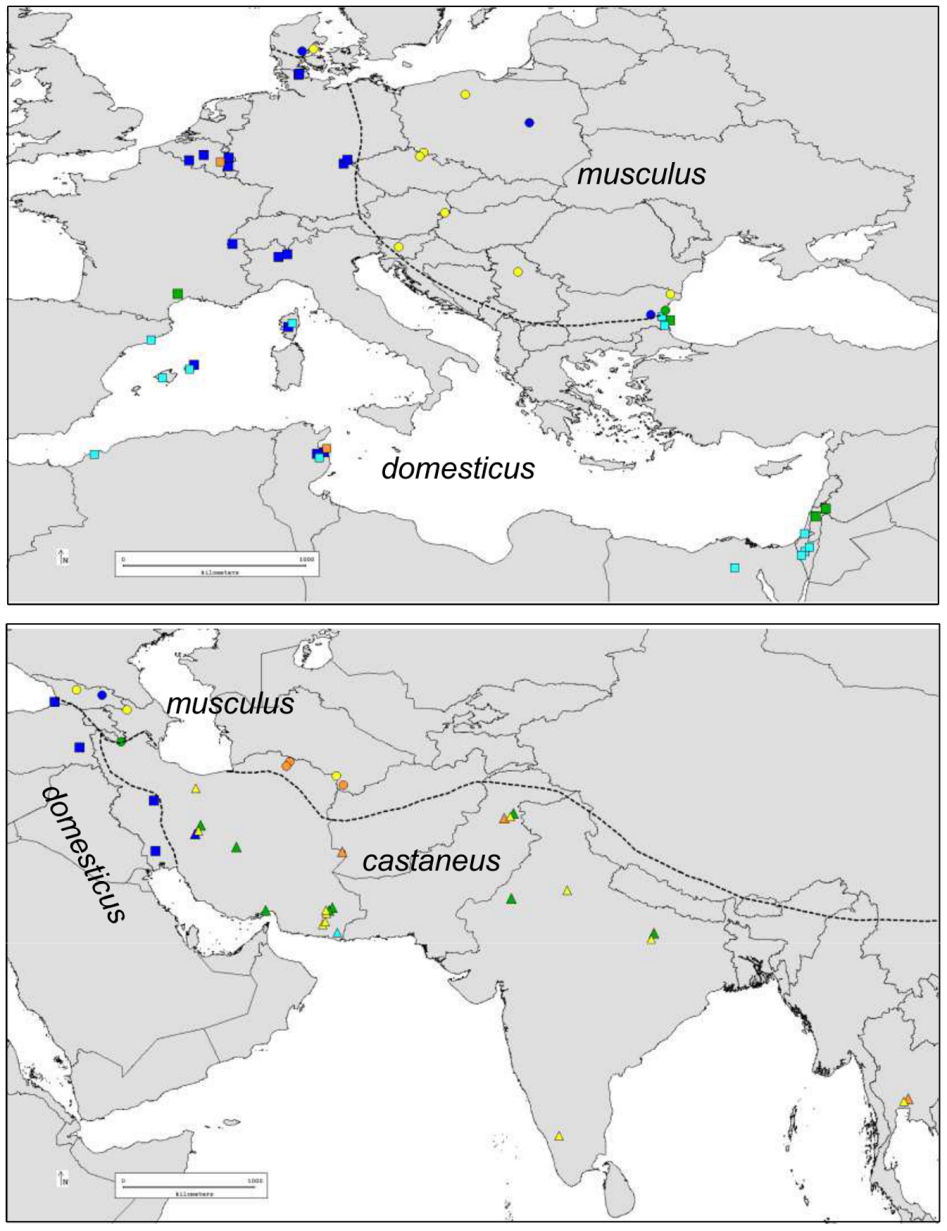
Geographic distribution of groups of alleles in the house mouse. The shape of the symbols indicates subspecies (square, *M. m. domesticus*, circles *M. m. musculus*, triangles *M. m. castaneus*). Colors indicate lineages as in Fig. 3.

The geographic distribution of the alleles reveals a clear separation of some of the house mouse pseudo-lineages we defined (Fig. 4). For instance, the dark blue and light blue lineages of *M. m. domesticus* predominate in NW Europe and around the Mediterranean, respectively. The yellow lineage is predominant in the European part of the distribution of *M. m. musculus*, while frequent in *M. m. castaneus* and geographically widespread, from Iran to SE Asia through India. The orange group of sequences is sporadic in the three subspecies. The green group is geographically widespread, frequent in *M. m. castaneus*, sporadic in *M. m. domesticus* and absent in *M. m. musculus* except close to the Bulgarian hybrid zone with *M. m. domesticus*. Note though that these orange and green categories do not represent well sustained phylogenetic entities, but rather collections of deeply branching alleles illustrating potential sharing of ancestral lineages among the subspecies.

### Characterization of a peculiar short Znf array in *M. spretus*

We were intrigued by the sequence with only two ZnF repeats that we found in a *M. spretus* specimen (sequence 20 in Fig. 2). In fact based on DNA sequence, only one of the two repeats appears as a putatively functional C2H2 zinc finger, and one may wonder how such a reduced ZnF domain could fulfill the DNA binding activity of PRDM9. The mouse genotyped by PCR that harbored this very short allele appeared heterozygous, with another allele that we also sequenced and has 9 repeats (allele 19, Fig. 2). This mouse belonged to early generations of a wild-derived colony maintained in the laboratory (strain SMZ) and we genotyped by PCR mice from later generations of that strain, after they had gone through 15 generations of brother-sister mating. All mice presented the short PCR band corresponding to a 2 ZnF domain, plus either one band (corresponding to 9 ZnFs) or two bands (9 and 12 ZnFs; Fig. S1A). A Southern blot analysis revealed a fragment (15–20 Kb) much larger than expected from the predicted restriction map of the *Prdm9* locus, along with fragments of sizes predicted from the restriction map and corresponding to the longer PCR alleles (9 and/or 12 ZnFs according to PCR genotype,Fig. S1B). The larger fragment was only observed in mice with the 2 ZnF PCR allele. The 2 ZnF allele therefore most likely represents a paralog of *Prdm9*, which appears to be transcribed (Fig. S1C) and translated (Fig. S1D) in testes. The occurrence of this paralog appears rare in nature as we detected it in only two mice among 34 wild mice captured during a single campaign in the locality of origin of the SMZ progenitors (not shown).

### Patterns of amino-acid variation among ZnF units

The 78 DNA alleles of the Zn finger domain translate into 75 different protein variants and contain 118 different DNA repeat sequences. These repeats are 84 bp long, with the exception of one harboring a 9 bp deletion and found at the first position of the array. Ten different repeats (including this shorter one) are always found at first position and were clearly distinct from the others not found at this first position. These first position repeats are predicted to be unlikely to have the function of a Zinc finger as they lack an essential cysteine required for Zinc binding [40]. Three repeats have stop codons and lie in last position of the array. We then examined amino-acid variation among the remaining putatively functional 105 DNA repeats.

Fifteen among the 28 codons of the ZnF unit show non-synonymous variability (Fig. S2 and S3A). Twelve variable positions show two or three variant amino acids. Some rare variants are common to specific taxa, such as amino acid Q at position 1, found in *M. spretus* alleles only. Similarly, amino acid S at position 16 is found only in *M*. *macedonicus* and *M*. *spicilegus* alleles but in all of them. In contrast, N at position 1 is found in half of the 75 protein variants, including *M. musculus* alleles of the three subspecies and *M. macedonicus* and *M*. *spicilegus* alleles. W at position -5 is present in most protein variants (63 out of 75) and always within the carboxy-terminal ZnF, excluding notably *Pyromys*, *M*. *famulus*, *M*. *cervicolor* and 4 out of 5 *M*. *spretus* alleles. This substitution is a C to T transition within a CpG and thus potentially prone to DNA methylation and mutation (the only CpG in the consensus of all repeats). Based on the structure of Zif268, a transcription factor containing three C2H2 ZnFs [40], position −5 is a residue with phosphate backbone contact.

The most diverse codons are at positions −1, 3 and 6 of the ZnF unit with nine, eight and five variant amino acids respectively. Each of these three codons (−1, 3 and 6) shows its own pattern of diversity (Fig. S3A). Position 6 is the least diverse with two major variant amino acids (Q and K) shared by all *Mus* subspecies and species and both contained in each of the 75 alleles. Position −1 presents three major amino acid variants with frequencies above 8% among the 1109 ZnF sequenced, two being shared by all groups of alleles (Q,V) and one (A) found in every allele of all groups except the distantly related *M*. *cervicolor*, *Pyromys* and *M*. *famulus* alleles. Amino acid at position 3 is the most diverse, with five major variants (D, N, V, H, S), and includes two variants shared by all alleles (D,N), one variant found only in *M. m. musculus* (V), or enriched in *M. macedonicus*/*M. spicilegus* (H) or in *M. spretus* (T).

Most of the variation seen between ZnF repeat units thus involves non-synonymous substitutions at the three amino-acid positions critical for the DNA binding activity of the ZnFs (positions −1, 3 and 6) as previously reported in rodents and primates [18]. This is highly suggestive that natural selection is favoring such variations, while other positions are either under purifying selection and/or homogenized by frequent copy number variation or occasional unequal recombination events inside the ZnF array. Some authors have attempted to give statistical support to the excess of non-synonymous differences at these key amino-acid positions. However the method used (PAML, [41]) cannot be applied because it relies on inferring mutation rates along a phylogenetic tree of the repeat unit variants. Because of the mutation processes (duplication/deletion and gene conversion) involved in the evolution of such repetitive sequences and the resulting concerted evolution between copies, it is impossible to meaningfully represent the evolution of the sequences of the repeats along a tree.

### Diversity and species-specificity of ZnF variants

In order to focus on variations most likely to be related with function, we reduced the sequence of the ZnF arrays to their states at these three most critical positions (−1, 3 and 6) in each ZnF. This resulted in 53 different possible combinations of amino-acids among all sequenced ZnF repeats. The frequencies of the 53 ZnF variants show a wide distribution, with 19 major ZnF units ranging in frequency from 17% to 1% and 20 rare ZnF units, found only once among 1109 ZnF units sequenced from the wild (Fig. S3B). These variants could be classified into three categories: (i) those shared by all mice, (ii) ZnF variants shared between *Mus musculus* alleles only and/or enriched in two groups such as *M. m. domesticus* and *M. m. musculus* or *M. m. musculus* and *M. m. castaneus* and (iii) those found nearly exclusively in one group. Each species or subspecies (for which more than 50 ZnFs were sequenced) has on average 20 distinct ZnF variants. All ZnF variants in *M. musculus* alleles are shared between the three subspecies except rare variants. However, some ZnF variants are enriched in *M. m. domesticus* and in *M. m. castaneus* respectively. In contrast, although they were less intensely sampled than the house mouse, *M. spretus* and *M. macedonicus/spicilegus* have relatively frequent variants not shared with the house mouse.

### Organization of Prdm9 ZnF array and polarized variability

Based on the inference of allele phylogeny we wanted to examine whether functional aspects of this ZnF domain were also phylogenetically or taxonomically structured. We wondered whether alleles related in the phylogeny tended to share common DNA recognition capabilities. Reducing the 75 protein variants to only positions −1, 3 and 6 of the potentially functional repeats (*i.e.* excluding repeats in first position and those with stop codons, which were always in last position of the domain) resulted in 73 different variants (Table S1).

We then considered groups of alleles based on the inferred phylogeny, and searched the longest amino-acid word shared among members of a given group. Using an exact match search, the longest common words occur in the yellow, light blue and *M. macedonicus- M. spicilegus* groups of sequences, but are only 6 amino-acids long, the equivalent of only two ZnFs (Table S3). Common word lengths are limited to 4 in the other groups. By relaxing the quorum to allow one sequence in a group not to possess the common word, we find 3 words of length 7 in the dark blue group, and a word of length 4 in the *M. spretus* group, thus there is very little conservation of stretches of repeats in the array, even among closely related alleles.

**Figure 5:**
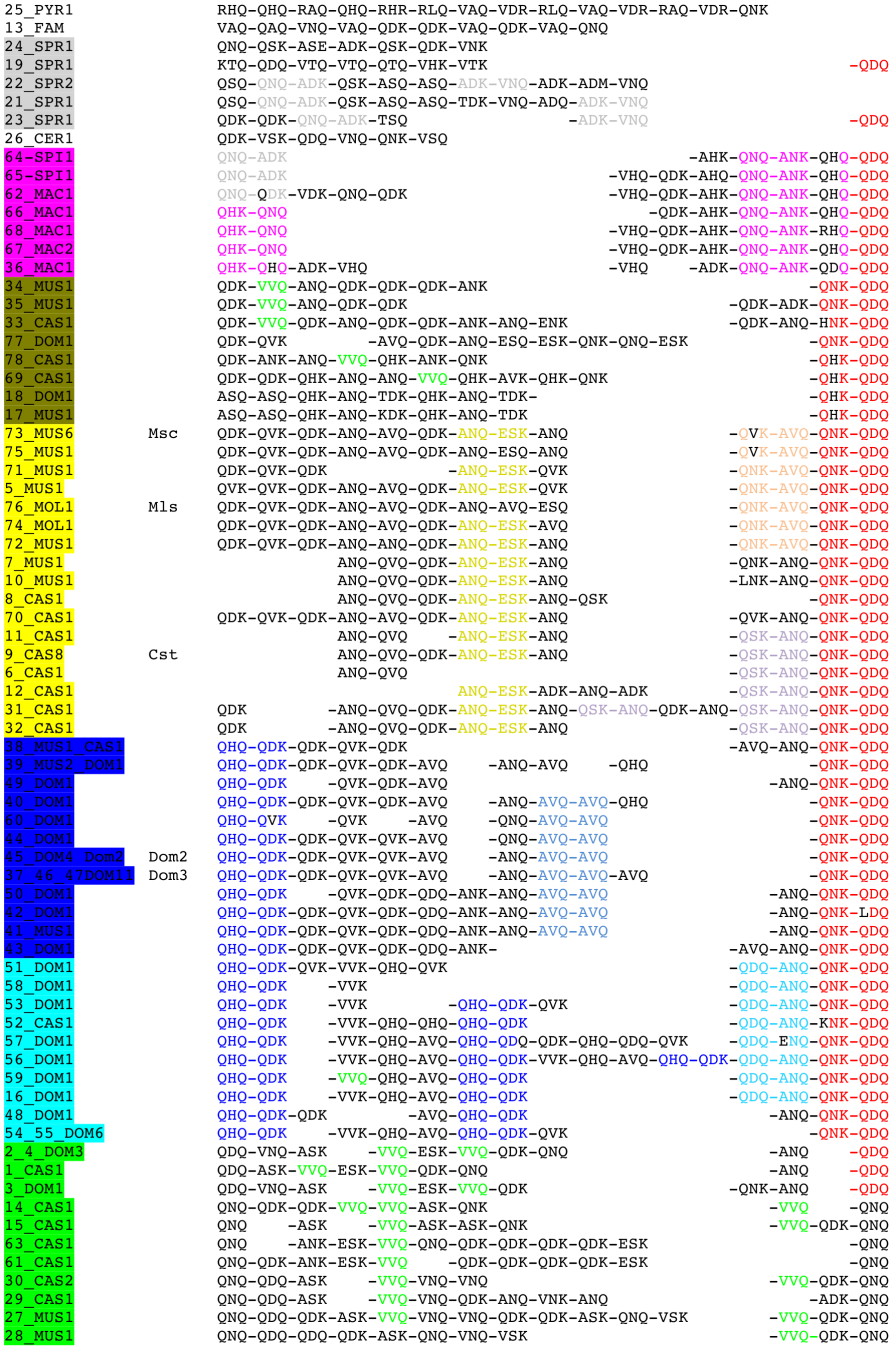
Simplified triplet protein variants of the *Prdm9* ZnF array in wild mice. Sequence identifiers are highlighted with colors as in the phylogenetic tree of DNA alleles in Fig. 2 and 3. Alleles of laboratory strains previously sequenced are identified as in [12]. Each ZnF is simplified to the three most variable codons −1, 3 and 6, and separated with a dash from the next ZnF. Sequences start at the first functional C2H2 ZnF (the second repeat) and end at the last carboxy-terminal ZnF of the protein. A few remarkable stretches of zinc fingers are highlighted: some are shared between most *M. musculus* protein variants (QNK-QDQ, red), some are shared between the twin species *spicilegus* and *macedonicus* (QNQ-ANK-**Q-QDQ, purple), some are shared between *castaneus* and *musculus* alleles (ANQ-ESK, yellow) and some others are specific or enriched in each of *domesticus* (QHQ-QDK, dark blue; AVQ-AVQ, light blue), *castaneus* (VVQ, green), *M. spretus* (ADK-VNQ; QNQ-ADK, grey); *M. macedonicus* (QHK-QNQ, purple) and *M. spicilegus* (QNQ-ADK, grey) groups of alleles.

We then used these common words to anchor the alignment of alleles inside the groups used to search them. We then attempted to extend the alignments from the anchors by relaxing the criteria in order to identify common and specific signatures in the different groups and to monitor their positions within the array (Fig. 5). Most (69%) of the 58 *M. musculus* triplet sequences share QNK-QDQ at the carboxy-terminal end of the protein, a pattern not found in any other non-*M*. *musculus* allele. The sequence QNQ-ANK-**Q-QDQ (where the asterisk means a polymorphic position) is shared between all 7 *M. macedonicus* and *M. spicilegus* alleles, at the carboxy-terminal end of the protein. *M*. *macedonicus* alleles show the specific QHK-QNQ sequence at the amino-terminal end of the array and *M. spicilegus* alleles share QNQ-ADK with *M*. *spretus*. Most *M. m. domesticus* sequences (18 out of 23) share QHQ-QDK at the amino-terminal end of the ZnF array (blue subtree). Most *M. m. domesticus* alleles originating from northern Europe are enriched within the carboxy-terminal half of the array with AVQ-AVQ (dark blue subtree in Fig. 3) whereas most of those originating from southern Europe and Mediterranean regions (light blue subtree) show QDQ-ANQ at the C term end and/or QHQ-QDK within the array. On the other hand, the distinction between *M. m. castaneus* and *M. m. musculus* alleles cannot be made without ambiguity, except within the yellow group, with *M. m. castaneus* alleles being distinguishable from *M. m. musculus* and *M. m. molossinus* alleles, each of these two subgroups showing a specific ZnF doublet within the carboxy-terminal half of the array. Among the more distantly related *Mus* species, although sample size is small, some unique features can be detected such as the presence of ADK-VNQ in three out of five *M. spretus* alleles, RAQ and RLQ for *M. pyromys* and the VAQ and QAQ for *M. famulus*.

### Distribution of predicted DNA binding motifs in the genome

We developed a different approach to address the question of the relationship between phylogeny and functional evolution. From the *Prdm9* DNA allele sequences (again retaining only potentially functional repeats) we predicted the DNA sequence recognized by the array of ZnFs, using the model of Persikov et al. [42] with a polynomial model (data not shown), and aligned these predicted motifs onto the mouse reference genome. Even if this model has limited predictability for protein domains including large number of ZnFs [43], we rationalized that it could be used as a tool to compare the alleles. We measured the overlap between predicted alignment hit coverage in the genome among *Prdm9* alleles and used this value to derive a distance between alleles (see Material & Methods). The clustering tree built from such a distance is shown in Fig. S4. There is a general tendency for alleles of the same phylogenetic group to cluster together, but internal branches are very short, and overall with this metric, intra-group differences are of the same order as inter-group because there is little overlap of hits among most pairs of alleles, so that the clustering signal is weak.

**Figure 6:**
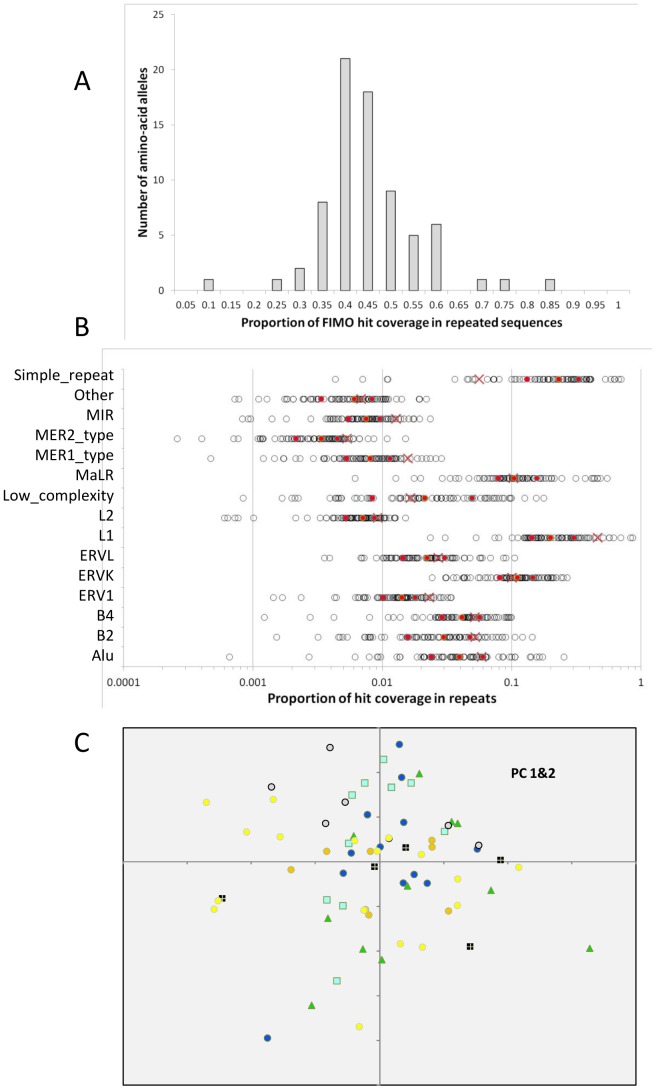
Predicted DNA binding sites of mouse *Prdm9* ZnF alleles and dispersed repeats. (A) Distribution among *Prdm9* alleles of the proportion of the coverage of hits of the predicted recognized DNA motifs that fall in dispersed repeated sequences, as annotated on the reference mouse genome. (B) Absolute proportion of hit coverage falling in a given repeat family for each of the sequenced allele. Red cross: expected proportion if hit coverage was proportional to the coverage of the family in the genome. Red circles: median, first and third quartile of the distribution across alleles. Note the log scales. (C) Projection of the alleles on the first two axes of the Principal Component Analysis on the relative proportion of hits of each allele in the different repeated families. Symbol colors refer to lineage colors as in Fig. 2 and 3. Symbol shapes are arbitrary. PC1 absorbs 35% of the variance, and PC2 13%.

Another way to analyze the predicted distribution of binding sites is to compare the identified genomic hits with given genomic features. We performed this analysis by measuring the overlap of predicted binding sites with the different families of interspersed repeated sequences, which indeed showed a large variation among alleles, with a minimum of 8% and a maximum of 80% for hit coverage in repeated sequences (Fig. 6A). The representation of different families of repeats among predicted hits shows extreme variations among alleles (Fig. 6B) with the hits of some alleles avoiding some repeat families, and those of other alleles falling quasi-exclusively in a given family. Overall, in proportion to their coverage in the genome, some families appeared over-represented (e.g. Simple repeats) and others under-represented (e.g. L1) among predicted hits (Fig. 6B). We used the relative importance of hit coverage of each allele in the different repeated families (considering only those hits that overlapped with repeated elements) to conduct a Principal Component Analysis. Fig. 6C shows that in such an analysis, there is no obvious grouping of alleles according to their clustering in the phylogeny, each lineage being widely spread across values of Principal components 1 and 2 (as well as for PC 1&3, and 2&3, not shown).

## Discussion

### Phylogeny, phylogeography and gene flow

One of the major motivations of the present study was to describe the variability and to infer the history of the diversification of *Prdm9* ZnF array among geographic populations of a given species, and between closely related taxa that extensively share polymorphisms and potentially still exchange genes. We have attempted to reconstruct the evolutionary history of the *Prdm9* ZnF arrays sequenced, which is challenging given its minisatellite structure. We used an alignment tool dedicated to such situations [44], and previously used with human and mouse minisatellites [45], [46]. Although the underlying model of this tool is a simplification of the complex turnover of these tandem repeats, which can occasionally imply complex conversion events and complex intra allelic rearrangements, it takes into account the major mode of mutation by simple indels, as shown experimentally for human *Prdm9* for instance [25]. Concerning nucleotide substitution, we tried different mutation weighing matrices for single base mutations, including a model accounting for differences in mutation rates between nucleotide positions, but found very little effect on the output alignment and clustering.

Most strikingly, we found that a fair amount of ancient polymorphisms appear to have been preserved for this gene, so that closely related taxa, such as *M. spicilegus* and *M. macedonicus*, or the house mouse subspecies, whose divergence times are similarly low [29], have not reached reciprocal monophyly. Among the house mouse subspecies, we have seen that the monophyly of *M. m. domesticus* cannot be formally rejected, but sharing of ancestral lineages is extensive between *M. m. musculus* and *M. m. castaneus*. This could result either from a recent divergence between *M. m. musculus* and *M. m. castaneus*, or from their secondary admixture in the past [47], [48]. The case of *M. spretus* would deserve further investigation as we found two extremely divergent lineages segregating in this species, with one of the alleles in the basal group being a paralog. Despite a certain degree of retention of polymorphism among house mouse subspecies, some alleles and lineages (or sublineages) are much more frequent in a given subspecies. The most striking situation is the blue lineage that appears to be typical of *M. m. domesticus*, and thus most probably diversified inside this subspecies. Another indication of this is the clear phylogeographic structure revealed in *M. m. domesticus* for this lineage, indicating further independent differentiation and diversification. A certain degree of phylogeographic structure over the range of *M. m. domesticus* was also documented for mitochondrial DNA and presumed to result from several routes of colonization of Western Europe and the Mediterranean basin [49]. The yellow lineage has also obviously diversified inside *M. m. musculus-castaneus*, but the effects of primary differentiation and secondary gene flow between these two subspecies are difficult to disentangle.

Our results can be compared to those obtained with four non-coding mouse hypervariable minisatellites, using many common wild mice DNA samples and the same alignment tool [46]. Sublineages also appeared to have diversified specifically in one or the other subspecies at these loci, but the general pattern was extensive lineage sharing between subspecies, as well as with *M. spretus*, and abundant evidence of exchanges between house mouse subspecies. Although the nature of the dataset (minisatellite variation) prevents quantification of these aspects, overall the pattern we obtain on *Prdm9* ZnF arrays appears more taxonomically structured. This characteristic could allow the emergence of hybrid incompatibilities between subspecies linked to *Prdm9* divergence, as described between certain combinations of *M. m. domesticus* and *M. m. musculus* genomes [26]. However, although hybrid male sterility is frequently observed between house mouse subspecies [50]–[54] the frequency of involvement of *Prdm9* in this phenotype remains to be assessed since the number of independent observations where it is known to play a role remains extremely limited [55]. Our dataset also gave evidence of recent gene flow of *Prdm9* across secondary hybrid zones or by long distance migration, as already inferred in genome-wide surveys [56], [57].

In any case, the emerging picture for the evolution of the ZnF domain of *Prdm9* in mice is not that of rapid phylogenetic differentiation through efficient lineage sorting, as would be expected for a gene submitted to directional divergent selection, a pattern often considered most likely to lead to hybrid incompatibilities [58], [59]. Overall our phylogenetic and phylogeographic data do not appear contradictory with the diversification of the *Prdm9* ZnF array being mainly driven by mutation, drift and demographic processes during the history of house mouse subspecies differentiation.

### Species-specific ZnF variants and polarized variability of the array

At the ZnF level, even reduced to the three most variable amino-acids, nearly all *M. musculus* ZnF variants are shared between the three subspecies although some are enriched in *M. m. domesticus* or in *M. m. castaneus*. In contrast, half of the *M. musculus* ZnF variants are not shared with *M. spretus* and one third are not shared with *M. macedonicus* and *spicilegus* (Fig. S3C), suggesting some specificity of variants at the level of species, even when closely related. Examination of stretches of ZnFs showed that blocks of two ZnFs distinguish species and even subspecies (Fig. 5). Most *M. musculus* protein variants share a specific ZnF doublet signature at the carboxy-terminal end of the protein as do *M. spicilegus* and *M. macedonicus* variants. The amino-terminal end of the ZnF array is more variable but still shows subspecies signatures. The central part of the array is the most variable. This polarized variability of the *Mus* ZnF array could be due to a higher turnover of the coding minisatellite in the central region of the array. Alternatively it could be the result of different modes of selection on the different parts of the array. Recent work dissecting the DNA binding specificity of a single *M. m. castaneus Prdm9* allele suggests that the positive selection pressure on carboxy-terminal ZnFs might be weaker because of weak DNA binding specificity [16]. In humans, the amino-terminal part of the ZnF array is the least variable but the analysis of *de novo* sperm mutant molecules of the *Prdm9* human minisatellite shows that rearrangements occur along the whole array without any distribution bias, supporting the hypothesis that selection rather than mutation modulates heterogeneous variability in the array. In addition, another source of modulation of *Prdm9* diversity suggested from molecular analysis in humans is the possibility of allele dependent rate of this minisatellite instability [25].

### Patterns of diversity of the three key amino acids binding DNA

**Figure 7:**
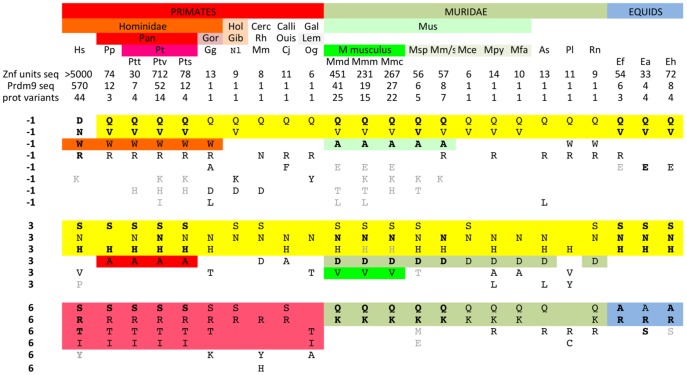
Patterns of diversity of amino-acids –1, 3 and 6 of PRDM9 ZnFs across taxa, species and subspecies. Number of ZnFs units sequenced, number of *Prdm9* ZnF arrays sequenced and number of protein variants are indicated for each species or subspecies of Primates, *Muridae* and Equids. Variant amino-acids at each of the three positions −1, 3 and 6 of the PRDM9 ZnFs are shown for each group. Variants present in every allele sequenced are in bold case and variants found in less than 10% of ZnF units are in grey case (all in normal case when one allele is available). Some variants at position −1 and position 3 are shared by most species (highlighted in yellow), others are shared by one taxon (highlighted according to the colour of the taxon). Hs: *Homo sapiens*; Pp: *Pan paniscus* (bonobo); Pt: *Pan troglodytes* (chimpanzee); Ptt*: P. T. troglodytes*; Ptv: *P. T. verus*; Pts: *P. T. schweininfurthii*; Gg: *Gorilla gorilla*; Hol: *Holobylata*e; Nl: *Nomascus leucogenys* (Gibbon); Cerc: *Cercopithecidae*; Mm: *Macaca mulata* (Rhesus monkey); Calli: *Callitrichidae*; Cj: *Callithrix jacchus* (Ouistiti); Gal: *Galagidae*; Og: *Otolemur garnettii* (Lemur); Mm: *Mus musculus*; Mmd: *Mus musculus domesticus*; Mmm: *M. m. musculus*; Mmc: *M. m. castaneus*; Msp: *Mus spretus*; Mm/s: *Mus macedonicus* and *spicigelus*; Mpy: *Mus Pyromys platythrix*; Mfa: *Mus famulus*; As: *Apodemus sylvaticus*; Pl: *Peromyscus leucopus*; Rn: *Rattus norvegicus*; Ef: *Equus ferus*; Ea: *Equus asinus*; Eh: *Equus hippotigris*. Data was gathered for *Mus* ZnFs from this study, for *Homo sapiens* ZnFs from [10], [12], [20], [23] for *Pan* ZnFs from [22], [79], for Equids from [80] and retrieved from GenBank for other individual alleles (Gg, Nl, Mm, Cj, Og, Apos, Perol, Rn).

When considering only the three codons −1, 3 and 6 of the 75 protein variants of *Mus*, 73 variants were found, illustrating that nearly all variability is contained within these three codons. Aside from these three codons, two sites (positions 1 and −5) show moderate variability and position 1 is also found variable in human *Prdm9*. The three key amino-acids for DNA binding show different patterns of diversity in *Mus*, position 6 being less diverse than positions −1 and 3. We questioned the generality of this finding by comparing our results to those available in Primates, *Muridae* and Equids (Fig. 7). A striking difference in the pattern of diversity between the three codons is observed. Position 6 shows a specific set of high frequency variants for each taxon with S, R, T and I found in Primates, A and R in all Equids and Q and K in *Muridae*. In contrast, positions −1 and 3 show two classes of amino acid variants, one being shared by nearly all species (Q and V at position −1; S, N and H at position 3) and the other being specific to one taxonomic group. We also note that position 2, known to be involved in DNA binding specificity, is not variable in *Mus* and is highly conserved in chimps and humans, *Muridae* and Equids. Interestingly, this residue is predicted to interact with a base complementary to the one in contact with position 6 from an adjacent zinc finger [40]. This structural property may thus add a constraint on the evolution of position 6. Whatever the underlying constraints, the different patterns of diversity of the three key amino acids for binding DNA result from distinct evolutionary turnover at the three codons. This suggests a model where new ZnF units are produced by changes at three variable amino acid positions subjected to distinct selection pressures, driving their diversification at different evolutionary timescales.

### What drives the evolution of *Prdm9*?

Frequent duplication/deletion of repeat units by recombination or replication slippage during the evolution of the *Prdm9* ZnF array should tend to homogenize the sequences of the different copies, and this effect is visible when comparing different species or lineages that have evolved independently [18]. However, non-synonymous differences at key codon positions controlling interaction of PRDM9 with DNA clearly escape such homogenization more than other sites, suggesting that some type of selection controls variation at these positions. The quantification of the effects of selection and the way in which it acts remain to be evaluated. The only mechanism that has been proposed is related to the predicted erosion of DNA recognition sequences by gene conversion events accompanying DSB repair (the so-called hotspot paradox, [60]–[63]). Indeed, there is evidence that, as compared to the chimpanzee, the human genome is depleted in recognition sites for the most frequent *PRDM9* allele, which could be the consequence of such an erosion process [11]. The more frequent and the older a given *Prdm9* allele, the more advanced such erosion of its specific DNA recognition sites, potentially to a point where they would become so rare that meiosis could be impaired. At some undetermined level of hotspot erosion, selection could favor new alleles with different recognition sites. The total number of DSBs per meiosis is of the order of 200–300 in mice and a two-fold reduction of DSBs is known to hamper synapsis between homologs and fertility [64]. In addition, all chromosomes are not equal with respect to homologous interactions and a unique situation involves the sex chromosomes. In the heterogametic sex, recombination and pairing between sex chromosomes are restricted to the PAR (Pseudo Autosomal Region) where the density of recombination is much higher than on autosomes. If erosion of *Prdm9* binding sites occurs, one may expect its strength and its consequence on homologous pairing to be greater than on autosomes. Thus the PRDM9 independency of hotspots in *M. m. domesticus* PAR [13] could be the result of such erosion. One should also note that, surprisingly, *Prdm9* seems to be absent or nonfunctional in several vertebrate phyla [19] and in particular in *Canidae* [65], [66].

An additional parameter of allelic diversity and possibly influencing fitness is the genomic distribution of recombination sites. The available data based on a few alleles in human and mice indicate very different sites associated with different *Prdm9* alleles [13], [17]. Using the available *in silico* prediction method, with its known limited prediction power, we have found little overlap of predicted binding sites between alleles (Fig. S4). Interestingly, analysis of GC content evolution at recombination hotspots mapped in the strain C57BL/6 from *M. m. domesticus* reveals a local increase in GC content [67]. This suggests that these hotspots, and thus the *Prdm9* alleles specifying their localizations, have been active long enough to impact on the genome content by the process of GC-biased gene conversion [68]. In our analysis, we found that the C57BL/6 *Prdm9* ZnF allele (allele 45 in Fig. 3B) is relatively frequent in *M. m. domesticus*, closely related to the most frequent allele in our sample (allele 46), and part of a group of related alleles (37, 39, 40, 44–47 and 60, Fig. 3B), that altogether represent a significant fraction of the diversity found in *M. m. domesticus*. The hotspots specified by this group of alleles may thus substantially overlap, which is also suggested by the analysis of predicted hits in the genome (Fig. S4).

Experimental data have suggested some relationship between the position of either historical hotspots or DSBs and genome annotation. In humans, potential recognition sites of the major European allele appear to be particularly active when located in a specific repeated element, THE1 [14], and a weaker penetrance effect was associated with some LINE subfamilies for West-African alleles [17]. In mice, some families of repeated elements such as the MalR family are overrepresented in hotspot regions of one allele tested [69]. Repeated elements have been speculated to be favored hotspot sites because they offer naturally abundant and dispersed targets in the genome, advantages that are however expected to be counterbalanced by the risks of rare harmful ectopic recombination [70]. There is also evidence of coevolution of ZnF proteins with interspersed repetitive elements at a broad phylogenetic scale [71]. We have attempted to characterize how repeated sequences are targeted by different mouse *Prdm9* alleles. We found extreme variability in the proportion of predicted targets overlapping with dispersed repeats. We also found extreme variability in the frequency distribution of targets among repeat families, with some alleles apparently “specialized” in a given family, but little relationship between this distribution and the inferred evolutionary distance between alleles. Given the allelic richness found at *Prdm9*, and the complexity of the relationship between sequence variation and DNA recognition specificity, it would be surprising that the mode of evolution of this gene be driven by a simple selection regime with a unique origin. It would appear more plausible that the diversity of targeted DNA motifs correspond to a variety of evolutionary origins and consequences of the selection pressure(s), eventually operating at different time scales. It cannot either be excluded that the high mutation rate of this gene domain represents a selective burden, and that purifying selection plays an important role in regulating allele frequencies. The association of a high rate of mutations with either deleterious or advantageous effects but on different traits, and of a mechanism limiting the lifetime of initially favored alleles draws an original and complex framework for the evolutionary dynamics of this gene, that will be important to further evaluate and quantify.

## Materials and Methods

### Wild mice samples

Table S2 gives the complete description of the wild mice used in this study for PCR typing and sequencing the *Prdm9* ZnF array. They represent several taxa within the genus *Mus*, with particular emphasis on subspecies of the house mouse (*Mus musculus*), but also including other closely related Palearctic species (*Mus spretus, M. spicilegus and M. macedonicus*) as well as more distantly related species used as outgroups. Although most samples were directly from the wild, some belonged to wild-derived colonies maintained in the laboratory (with various levels of inbreeding), in which case the strain name is indicated in Table S2. All animal procedures were performed under the permission of the French authorities (Permit number C34-172-23), under the control of the Ethics Comity of Université Montpellier 2 and the protocols validated by the Ethics Committee for Animal Experimentation (CEEA-LR-11028).

### PCR genotyping and sequencing of *Prdm9* Znf minisatellite

The Znf arrays of each of 250 mice was PCR amplified from 20 ng of genomic DNA in 10 ul reaction of the PCR buffer (named here AJ Buffer) described elsewhere [72] and with 0.5 uM of primer mZPrdm9-F1 (GAGAATTTGCAATGGGGCTTT) and primer fl1500U20 (ATATGGAATGGAATCATCGC). Cycling conditions were: 96°C 30 s followed by 28 cycles including 96°C 10 s, 55°C 20 s and 70°C 2 min. Agarose gel electrophoresis (2%, Seakem) revealed the sizes of the alleles and amplification was performed again from a subset of mice, scale up to a 50 ul reaction and for 30 cycles depending on allele sizes. Bands were purified from agarose gels using the Qiagen Gel purification kit and the amount of DNA recovered was estimated by gel electrophoresis. Sanger sequencing was performed from each end of the PCR product with the Big Dye Applied Biosystems sequencing kit from 50 ng of purified PCR product using primer Meis284L23 (ATTGTTGAGATGTGGTTTTATTG) or primer mZPrdm9-R1 (GGCCAGACAACAAATACAGA). Subcloning using the TOPO TA cloning system (Invitrogen) was performed for a subset of PCR products, either larger than 14 repeats or when the two alleles were close in size and could not be efficiently separated on gels. Most PCR products could not be sequenced up to their ends in both directions. Based on accurate estimates of fragment sizes on gels, assembly of the forward and reverse sequences was made possible despite the repetitive nature of the region.

### Southern blot, RT-PCR and western blot analyses

Southern blot: Twelve μg of Apal1 genomic DNA digests were run in a 0.7% agarose gel and transferred onto a nylon membrane. Seventy ng of a 360 bp PCR product overlapping the intron/exon10 junction of *Prdm9* (primers: CCTCTGCCTGGGTTTGGATT and AGCTGGGTGTGCCTTAACTC; coordinates GRCm38: 15545119-15545479) was P32 labeled (Prime-a-Gene Labelling System, Promega), hybridized to the nylon membrane in 0.25 M Na2HPO4, 7% SDS, 10 g/L BSA at 65°C, washed in 0.02 M Na2HPO4, 1% SDS at 65°C and exposed to a phosphor screen (Molecular Dynamic).

RT-PCR: mRNA was extracted from mouse testes using the GenElute Mammalian Total RNA Miniprep kit (Sigma) as recommended by the manufacturer. Five hundred ng of mRNA was reverse transcribed in 20 µl using 200 units of super script 3 reverse transcriptase (Invitrogen kit), after digestion of residual DNA (kit DNA free, Ambion). One μl was subsequently subjected to PCR in 10 µl AJ buffer using Taq:Pfu (0.5U∶0.05U), 0.5 µΜ of primer Meis2848L23 (exon 10; ATTGTTGAGATGTGGTTTTATTG) and either primer fl1075U21 (exon 7; ATCTGATCTACCAGTCGGTCT), or primer Pr347U18 (exon2/exon1 junction; CCCAAGGTCAAAGATGAA), or primer Pr274U18 (exon1; GTCCTGCACCATGAACAC). Amplification was for 94°C 2 min followed by 35 cycles of 94°C 30 sec, 57°C 30 sec, 68°C 3 min plus a final step at 68°C 7 min.

Western blot: Seventy five μg of proteins extracted from adult mouse testes were run into a precast 10% acrylamide gel (Biorad) and transferred onto a PVDF membrane using the BioRad turbo transfer system for 7 min. The membrane was hybridized in TBST 1X 0,5% milk buffer with a primary in house rabbit antibody raised against mouse PRDM9 and with the secondary anti-Rabbit HRP (Jackson, ref 711-035-152) diluted 1/5000. Luminol revelation was performed using the Supersignal West Pico Chemiluminescent Substrate (Thermo Scientific) and exposed for 30 min to Amersham Hyperfilm ECL.

### Building phylogenetic trees from the minisatellite sequences

The tandemly repeated minisatellite structure of the *Prdm9* ZnF array is prone to high levels of repeat copy number variation, preventing the use of classical multiple alignment methods to compare alleles. We thus resort to an evolutionary model that accounts for both duplication/deletion of repeats, and for point mutations/indels inside the repeat sequences. For this, we combined the use of a Tandem Repeat specific alignment method, MS_Align [45], with a distance matrix between the individual repeat sequences. An all-against-all allele comparison with this system yielded a matrix of distances between allele sequences, from which we could infer an evolutionary tree using a minimum evolution approach, FastME [73] and estimate confidence values for internal nodes using the Qualitree program [74] from which we report the Rate of elementary well-designed quartets (Re) values at the nodes of the trees as measures of confidence levels in these nodes (note that regarding confidence values, bootstrapping is prevented by the prevalence of gaps in a multiple alignment).

Measuring sequence similarity between allele sequences with MS_Align requires first to compute an evolutionary distance matrix between the individual repeat sequences, and second to estimate the penalties charged for the duplication/deletion of a repeat within the alleles. We detail these two aspects, before explaining how the reliability of the inferred tree was evaluated.

1. **Distance between individual repeat sequences.** We performed multiple alignments of the repeat sequences (with Muscle) and obtained a clear separation between those in the first array position and all other repeats. The 118 distinct individual repeat sequences were partitioned in two groups based on sequence proximity: on one hand, the ten repeats occurring at the first position in the array, and the 108 remaining repeats. In each group, the repeat sequences are highly similar, but differ markedly from those of the other group. This partition in two groups is also supported by functional arguments because the first repeat is not predicted to be a functional ZnF by current models, and it evolves differently from the others. Based on the multiple alignment of repeats in each group, we selected the best nucleotide substitution model by fitting classical evolution model for each group using the MEGA program [75]. The Jukes and Cantor model turned out to be appropriate for the first position repeats, and we counted as a single mutation the 9 bp deletion found in one of the repeats in this group. In the second group of repeats, the Kimura 2-parameters with a gamma distribution of mutation rates among sites (Gamma parameter = 0.31286) obtained the best fit. Which distance was used in the comparison between these two groups of repeats did not change the results (i.e. when computing distance between alleles), since they were clearly separated whatever the model used, making the transition from one type to the other impossible at the evolutionary scale considered here.
2. **Aligning the alleles and inferring the tree.** MS_Align computes an optimal alignment between two repeat unit sequence when considering the events of changing one unit to another (penalty M, stands for mutation), duplicating one unit in tandem (penalty A, for Amplification) or deleting one unit in tandem (penalty C, for contraction). The mutation penalties (M) depend on the repeats involved and were given by the above-mentioned distance matrix between individual repeats in which each was multiplied by a fixed coefficient and rounded to an integer. We chose a symmetric model (A = C = 1) and tried several coefficients, which impacts the penalty ratio between mutation/duplication. We compared the alleles with MS_Align version 2 [45], inferred a tree from the inter-allelic distances, and evaluated the tree reliability using Qualitree. We chose the tree having the best VAF measure (Variance Accounted For, see below): the one that best represents the evolutionary distances between the alleles. It was the tree obtained with alignment penalties A = 1, C = 1, and coefficient 1000, with a VAF of 0.96 and an average Rate of well designed quadruples (Re) over all internal nodes equal to 0.85.
3. **Evaluating the confidence in the tree.** Qualitree computes the percentage of Variance Accounted For (VAF) for the whole tree, and the Rate of elementary well-designed quartets (Re) for internal nodes. VAF measures the concordance between the inter-allelic distances in the matrix and those on the tree to evaluate how well the tree reflects the evolutionary relatedness between its “taxa”. Re estimates how well an internal node is supported by the split of all possible quartets of taxa going through this node (see [74] for the mathematical formulas). We report Re at the nodes of the trees in the figures.

### Searching for common words between Zinc Finger arrays triplet amino-acid sequences

The sequence of each ZnF repeat was summarized by the triplet of amino-acids at positions -1, 3 and 6 according to the C2H2 ZnF nomenclature. Thus each *Prdm9* allele is represented by an ordered series of as many such triplets as it possesses functional ZnFs. For the major groups of alleles appearing in the inferred phylogeny, we searched for the longest exact common words that are shared between their triplet sequences with the program RISO[76]; no substitution was allowed and a quorum of 100% was required. The size of the longest common words captures the similarity within each group and thus, their length gives a rough measure of this similarity. These triplet protein variant sequences were aligned using these common words as anchors inside each of the phylogenetic subgroups in which they were determined. Common words could be extended by eye to common stretches of ZnF units, allowing arbitrarily not more than one degenerate site over 6 amino acids. Common signatures shown may not represent all possible shared groups of ZnF units.

### Prediction of DNA motifs recognized and related analyses

We used the online software (http://zf.princeton.edu/) based on the method of Persikov et al. [42] using the SVM polynomial model to predict DNA motifs recognized by each of the amino-acid sequence alleles of the ZnF arrays. We then searched for matches of the predicted DNA motifs when aligned without gaps along the mouse genome (build mm9), using FIMO (http://meme.nbcr.net/meme/cgi-bin/fimo.cgi, [77] with an arbitrary P-value threshold of 10^−4^. This produced for each allele a series of hits in the genome. We then compared pairs of alleles by calculating the following distance:

D = 1-(Hit_intersection/Hit_Union),

where Hit_intersection is the genome coverage (in bp) of the intersection of the FIMO hits of the two alleles compared in the genome and Hit_Union the coverage of their union. We then used the matrix of pairwise distances between alleles to build a clustering tree with the neighbor-joining algorithm, as implemented in software MEGA [75]. We also determined the overlap between the FIMO hits (after concatenating overlapping such hits) of each allele and the repeat sequences annotations of the mouse genome. We only considered overlaps at least as long as the whole length of the predicted motif (3× the number of ZnFs). From this and for each allele we could calculate the base pair coverage of the FIMO hits lying in different families and subfamilies of repeated elements. Using the proportions of the coverage in repeats lying in the different repeat subfamilies for each allele, we ran a Principal Component Analysis (function ‘princomp’ in the R development package, [78]) with repeat families as variables and alleles as observations. In this analysis, each *Prdm9* allele is thus characterized by the partition among the different repeat families of the coverage of its predicted DNA recognition motifs falling in repeat families.

## Supporting Information

Figure S1 **A functional paralogous copy of** ***Prdm9*** **contains two ZnFs in a** ***Mus spretus*** **population.** (A) PCR genotyping of *Prdm9* ZnF array in B6 and in *Mus spretus* derived lines SEG, SFM and SMZ. Four SMZ individuals were genotyped with primer mZPrdm9-F1 and primer fl1500U20. (B) Southern blot analysis of B6, SEG, SFM and SMZ individuals using ApaLI as restriction enzyme and a 360 bp *Prdm9* probe overlapping the stop codon of the last exon (coordinates GRCm38: 15545119- 15545479). (C) RT-PCR transcription analysis from B6 and SMZ testes mRNA. PCR amplification from reverse transcribed testes mRNA of B6 and SMZ using primer AA, close to the stop codon of last exon of *Prdm9* and primer 1075 in exon 7 (1), or primer AA and primer 347 overlapping exon1 and exon 2 (2), or primer AA and primer 247,close to the transcription start site. Observed sizes of RT-PCR products in SMZ correspond with a gene containing 2 ZnFs for the shortest and more intense band. (D) Western blot analysis of SMZ, B6 and *Prdm9^−/−^* testes protein extracts. SMZ shows a band corresponding to a 9 ZnF protein variant plus a supernumerary band, shown with an arrow, at the expected size for a 2 ZnF protein variant (64 KD).
(PDF)

Figure S2 **Amino-acid diversity along the PRDM9 ZnF unit.** The consensus, 28 amino acid long, *Mus* ZnF unit is shown (red letters). Numbering of amino acids positions respect the C2H2 ZnF nomenclature and is shown above the consensus. Variant amino acids found in all *Mus* alleles analyzed are shown below each position. Green highlighting: two cysteines and two histidines define a functional C2H2 ZnF. Grey letters: found in less than 1% of the 795 ZnFs units found among 75 protein variants. Black letters: between 1 and 10%. Yellow highlighting: around 10%; found in more than half alleles. Blue highlighting: found in every allele. Pink highlighting: private variant of *M. macedonicus/spicilegus*. Light brown highlighting: private variant of *M. spretus*.
(PDF)

Figure S3 **Diversity of PRDM9 ZnF variants between** ***Mus*** **species and subspecies.** (A) Diversity of variant amino-acids at each of the three key positions for DNA binding. Variant amino acids found in the 1109 ZnFs sequenced are highlighted in shades of grey for each of position −1, position 3 and position 6 of the ZnF unit in each group of mice, according to their frequency in each group. Variants found in every allele of the group are boxed. (B) Fifty three different ZnF units defined by variations at the three key positions −1, 3 and 6 were found among 1109 ZnFs sequenced. ZnF units are shown highlighted in shades of grey accordingly to their frequency in each group, including rare (pale grey; <2%), common (intermediate grey; 2%–10%) and frequent variants (>10%). ZnF units enriched in or specific of one group are highlighted accordingly to the color of the group.
(PDF)

Figure S4 **Phylogeny of** ***Prdm9*** **ZnF domain DNA alleles based on the comparison of the hits of their predicted recognized DNA motifs in the reference mouse genome.** Allele numbers and coloring are as in Figs. 2 and 3. Neighbor-joining trees are built from the raw pairwise distance (A, see text for the definition of the distance) or on its log-transform (B).
(PDF)

Table S1 *Prdm9* Zinc finger genotypes, DNA sequences, DNA alleles, protein variants and protein variants simplified to positions −1,3 and 6 of each zinc finger. The number of distinct DNA alleles, protein variants and “-136AASeq” in each subspecies or species is shown. Since a few are found in more than one subspecies or species the total exceeds the overall number of distinct DNA alleles (78), protein variants (75) and “-136AASeq” (73).
(DOCX)

Table S2 Samples used for genotyping.
(XLSX)

Table S3 Longest common stretches of ZnF variants in each group of alleles as defined by the phylogenetic trees of Figs. 2 and 3.
(XLSX)
