# Supplementary figures and images for: Diversity of Prdm9 Zinc Finger Array in Wild Mice Unravels New Facets of the Evolutionary Turnover of this Coding Minisatellite
Source: PLoS One. 2014 Jan 13;9(1):e85021. doi: 10.1371/journal.pone.0085021 (PMC3890296; doi:10.1371/journal.pone.0085021)

A

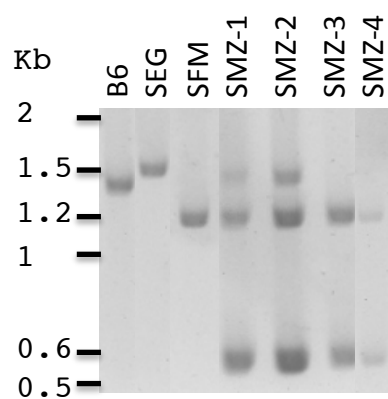

B

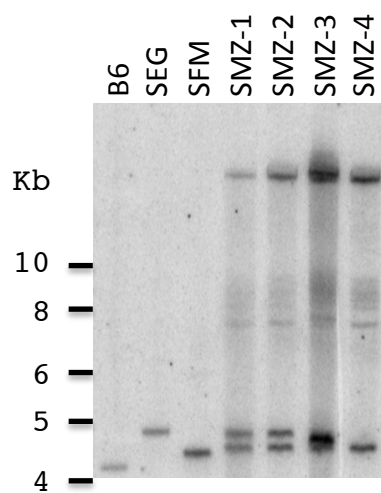

C

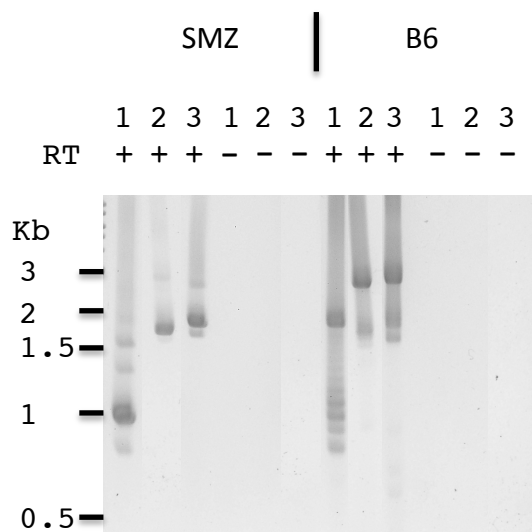

D

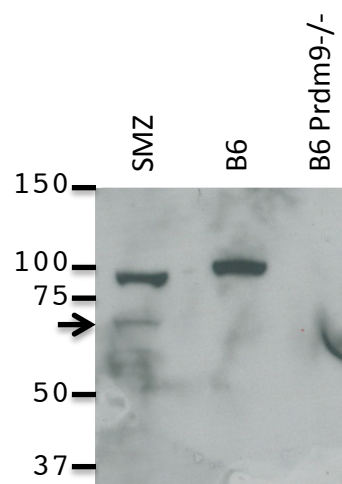

Supplement: Figure S1 — A functional paralogous copy of Prdm9 contains two ZnFs in a Mus spretus population. (A) PCR genotyping of Prdm9 ZnF array in B6 and in Mus spretus derived lines SEG, SFM and SMZ. Four SMZ individuals were genotyped with primer mZPrdm9-F1 and primer fl1500U20. (B) Southern blot analysis of B6, SEG, SFM and SMZ individuals using ApaLI as restriction enzyme and a 360 bp Prdm9 probe overlapping the stop codon of the last exon (coordinates GRCm38: 15545119- 15545479). (C) RT-PCR transcription analysis from B6 and SMZ testes mRNA. PCR amplification from reverse transcribed testes mRNA of B6 and SMZ using primer AA, close to the stop codon of last exon of Prdm9 and primer 1075 in exon 7 (1), or primer AA and primer 347 overlapping exon1 and exon 2 (2), or primer AA and primer 247,close to the transcription start site. Observed sizes of RT-PCR products in SMZ correspond with a gene containing 2 ZnFs for the shortest and more intense band. (D) Western blot analysis of SMZ, B6 and Prdm9−/− testes protein extracts. SMZ shows a band corresponding to a 9 ZnF protein variant plus a supernumerary band, shown with an arrow, at the expected size for a 2 ZnF protein variant (64 KD). (PDF) [file pone.0085021.s001.pdf]

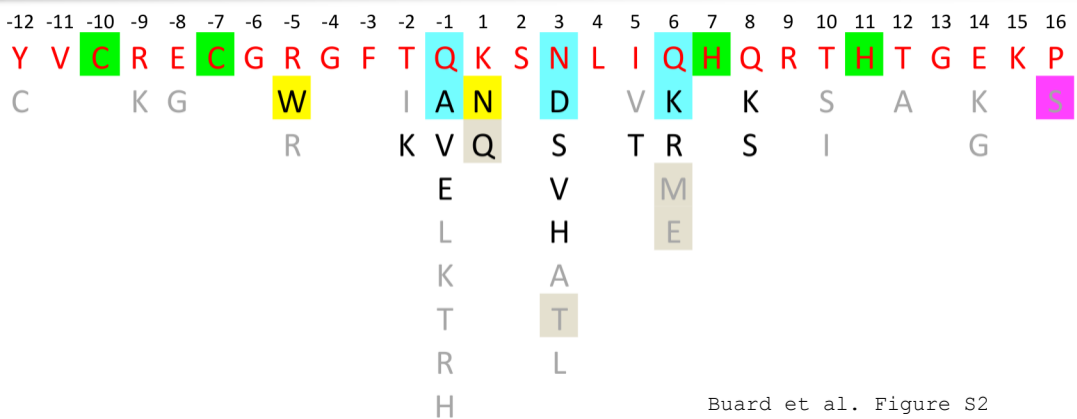

Supplement: Figure S2 — Amino-acid diversity along the PRDM9 ZnF unit. The consensus, 28 amino acid long, Mus ZnF unit is shown (red letters). Numbering of amino acids positions respect the C2H2 ZnF nomenclature and is shown above the consensus. Variant amino acids found in all Mus alleles analyzed are shown below each position. Green highlighting: two cysteines and two histidines define a functional C2H2 ZnF. Grey letters: found in less than 1% of the 795 ZnFs units found among 75 protein variants. Black letters: between 1 and 10%. Yellow highlighting: around 10%; found in more than half alleles. Blue highlighting: found in every allele. Pink highlighting: private variant of M. macedonicus/spicilegus. Light brown highlighting: private variant of M. spretus. (PDF) [file pone.0085021.s002.pdf]

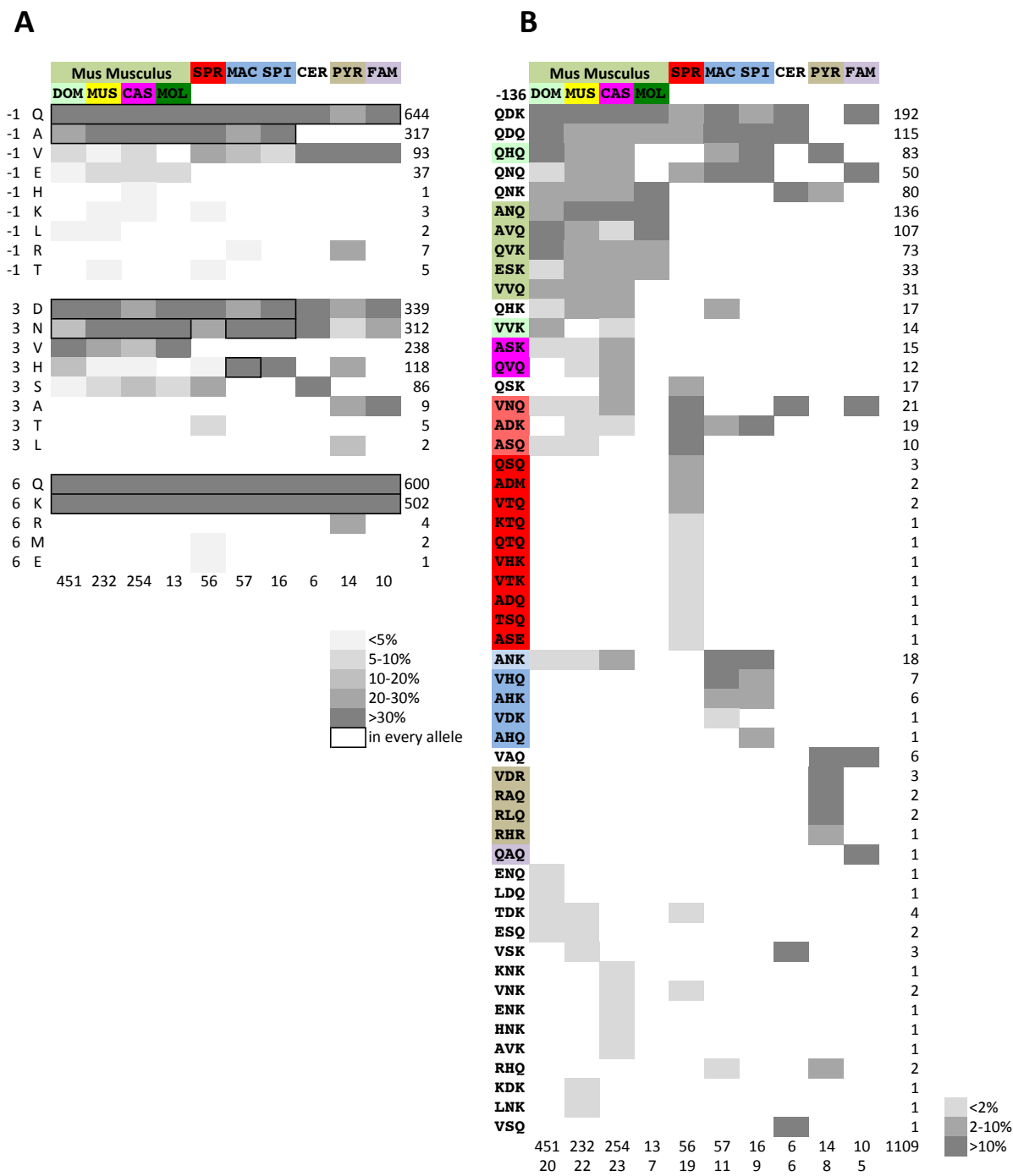

Supplement: Figure S3 — Diversity of PRDM9 ZnF variants between Mus species and subspecies. (A) Diversity of variant amino-acids at each of the three key positions for DNA binding. Variant amino acids found in the 1109 ZnFs sequenced are highlighted in shades of grey for each of position −1, position 3 and position 6 of the ZnF unit in each group of mice, according to their frequency in each group. Variants found in every allele of the group are boxed. (B) Fifty three different ZnF units defined by variations at the three key positions −1, 3 and 6 were found among 1109 ZnFs sequenced. ZnF units are shown highlighted in shades of grey accordingly to their frequency in each group, including rare (pale grey; <2%), common (intermediate grey; 2%–10%) and frequent variants (>10%). ZnF units enriched in or specific of one group are highlighted accordingly to the color of the group. (PDF) [file pone.0085021.s003.pdf]

Buard et al. Figure S4

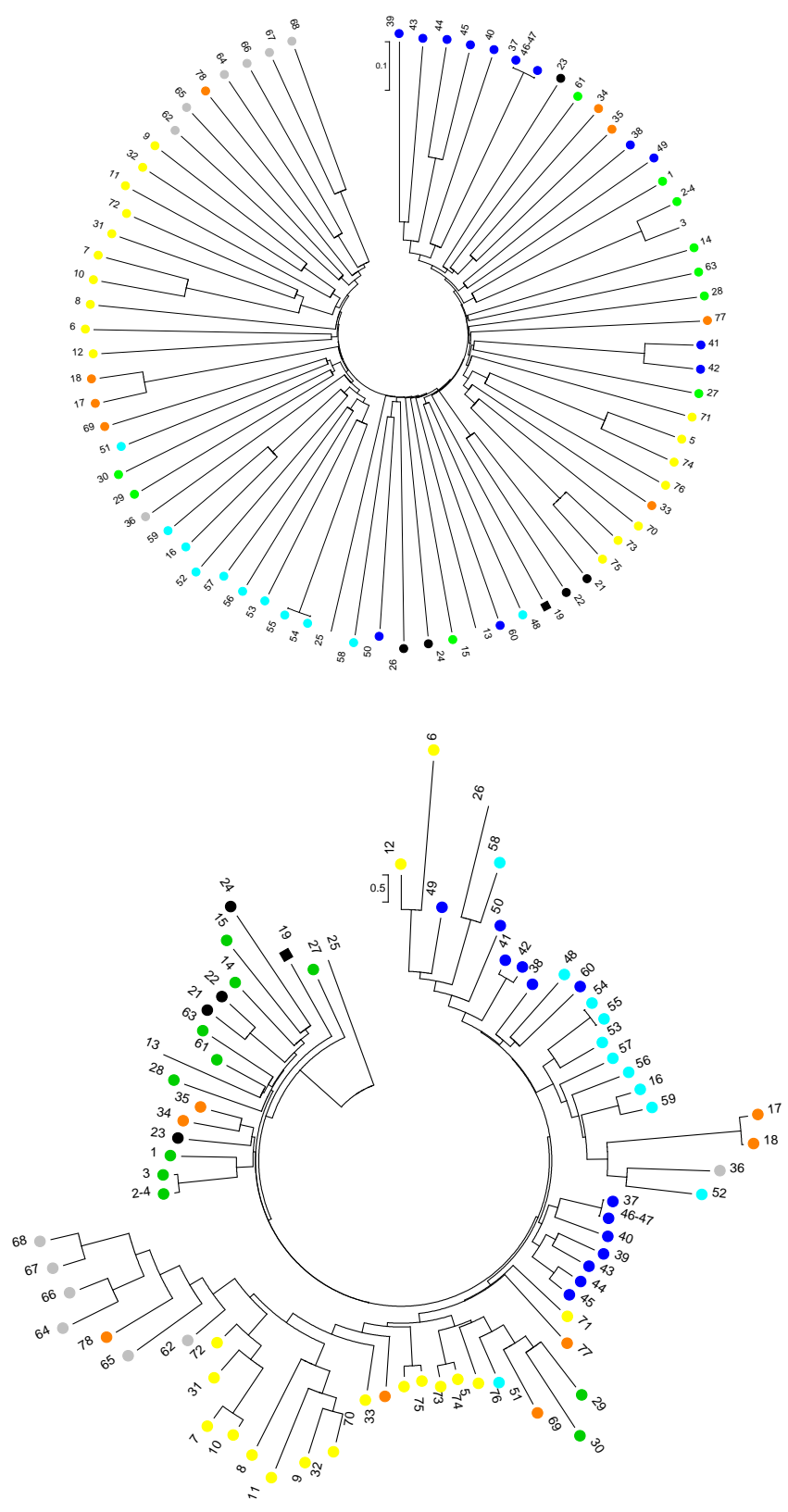

Supplement: Figure S4 — Phylogeny of Prdm9 ZnF domain DNA alleles based on the comparison of the hits of their predicted recognized DNA motifs in the reference mouse genome. Allele numbers and coloring are as in Figs. 2 and 3. Neighbor-joining trees are built from the raw pairwise distance (A, see text for the definition of the distance) or on its log-transform (B). (PDF) [file pone.0085021.s004.pdf]
