# Supplementary material for: Diversity of Prdm9 Zinc Finger Array in Wild Mice Unravels New Facets of the Evolutionary Turnover of this Coding Minisatellite
Source: PLoS One. 2014 Jan 13;9(1):e85021. doi: 10.1371/journal.pone.0085021 (PMC3890296; doi:10.1371/journal.pone.0085021)
Supplement: Table S1 — Prdm9 Zinc finger genotypes, DNA sequences, DNA alleles, protein variants and protein variants simplified to positions −1,3 and 6 of each zinc finger. The number of distinct DNA alleles, protein variants and “-136AASeq” in each subspecies or species is shown. Since a few are found in more than one subspecies or species the total exceeds the overall number of distinct DNA alleles (78), protein variants (75) and “-136AASeq” (73). (DOCX) [file pone.0085021.s005.docx]

|  | Genotypes (PCR) | DNA sequences  + labstrains | DNA alleles | Protein variants | -136 AA seq |
| --- | --- | --- | --- | --- | --- |
| *Mus musculus domesticus* | 81 | 42 + 2 | 27 | 25 | 23 |
| *Mus musculus musculus* | 67 | 19 + 1 | 16 | 15 | 15 |
| *Mus musculus castaneus* | 70 | 27 + 1 | 20 | 20 | 20 |
| *Mus musculus molossinus* | 1 | 1 + 2 | 3 | 3 | 3 |
| *Mus musculus spp.* | 4 |  |  |  |  |
| *Mus spretus* | 8 | 7 | 6 | 6 | 6 |
| *Mus macedonicus* | 8 | 6 | 5 | 5 | 5 |
| *Mus cypriacus* | 3 |  |  |  |  |
| *Mus spicilegus* | 2 | 2 | 2 | 2 | 2 |
| *Mus famulus* | 1 | 1 | 1 | 1 | 1 |
| *Mus caroli* | 2 |  |  |  |  |
| *Mus cervicolor* | 1 | 1 | 1 | 1 | 1 |
| *Mus pahari* | 1 |  |  |  |  |
| *Mus Pyromys platythrix* | 1 | 1 | 1 | 1 | 1 |
| TOTAL | 250 | 113 | 82 | 79 | 77 |
